# Baseline Susceptibility of *Spodoptera frugiperda* Populations Collected in India towards Different Chemical Classes of Insecticides

**DOI:** 10.3390/insects12080758

**Published:** 2021-08-23

**Authors:** Mahesh Kulye, Sonja Mehlhorn, Debora Boaventura, Nigel Godley, Sreedevi Kodikoppalu Venkatesh, Thimmaraju Rudrappa, Tara Charan, Dinesh Rathi, Ralf Nauen

**Affiliations:** 1 Crop Science Division, Bayer AG, Bangalore 560045, India; mahesh.kulye@bayer.com (M.K.); sreedevi.kv@bayer.com (S.K.V.); thimmaraju.rudrappa@bayer.com (T.R.); 2 Crop Science Division, R&D, Bayer AG, 40789 Monheim, Germany; sonja.mehlhorn@bayer.com (S.M.); debora.duarteboaventura@bayer.com (D.B.); nigel.godley@bayer.com (N.G.); 3 CoWrks India Private, Bayer AG, New Delhi 110037, India; tara.charan@bayer.com (T.C.); dinesh.rathi@bayer.com (D.R.)

**Keywords:** fall armyworm, insecticide resistance, resistance management, pyrethroid, corn, maize

## Abstract

**Simple Summary:**

Fall armyworm (FAW) is a highly destructive moth pest and its larvae feed on many different host plants, including major crops such as maize. This pest is native to the Americas but invaded the Eastern hemisphere in 2016. It is highly migratory and was first detected in India in 2018, where it rapidly became a major threat to maize production across India. FAW control mostly relies on the application of chemical insecticides and transgenic crop plants expressing insecticidal proteins of bacterial origin. FAW has developed resistance against insecticides, and for management purposes, it is important to assess its sensitivity status against different chemical classes of insecticides, particularly in invaded regions. In this study, we conducted more than 400 bioassays with nine different insecticides from seven mode-of-action groups on 47 FAW populations collected in 2019 and 2020 across different geographical areas in India. The baseline susceptibility determined for all these insecticides will help to set-up appropriate resistance management strategies to keep FAW infestations in India below economic damage thresholds.

**Abstract:**

Fall armyworm (FAW), *Spodoptera frugiperda*, is a major pest of maize in the Americas and recently invaded the Eastern hemisphere. It was first detected in India in 2018 and is considered a major threat to maize production. FAW control largely relies on the application of chemical insecticides and transgenic crops expressing *Bacillus thuringiensis* insecticidal proteins. Assessing FAW resistance and insecticide susceptibility is a cornerstone to develop sustainable resistance management strategies. In this study, we conducted more than 400 bioassays to assess the efficacy of nine insecticides from seven mode-of-action classes against 47 FAW populations collected in 2019 and 2020 across various geographical areas in India. The resistance status of the field-collected populations was compared to an Indian population sampled in 2018, and an insecticide susceptible reference population collected in 2005 in Brazil. Low to moderate resistance levels were observed for thiodicarb, chlorpyriphos, deltamethrin, chlorantraniliprole and flubendiamide in several populations (including the reference population collected in 2018). The highest resistance ratios were observed for deltamethrin which likely compromises recommended label rates for pyrethroid insecticides in general. Our data provide a useful baseline for future FAW resistance monitoring initiatives and highlight the need to implement insecticide resistance management strategies.

## 1. Introduction

Fall armyworm (FAW), *Spodoptera frugiperda* (Lepidoptera: Noctuidae), native to tropical and subtropical regions of the American continents has recently invaded the eastern hemisphere and is now widespread across the globe [1]. Since 2016, FAW expanded globally and was first reported in Africa [2,3], then spread to Southeast Asia [4,5] and Asia-Pacific, including Australia [6]. Its successful spread was attributed to several factors such as high reproductive capacity, long-distance migration, and polyphagy [7,8].

FAW was first detected in India in 2018 in maize fields of Karnataka state [4], and quickly invaded other regions in India [9]. FAW is a highly destructive polyphagous crop pest with the greatest yield losses projected in maize [10,11]. In India, maize is a staple food, and its production was estimated to be 28.6 million tons in 2019 [12]. The Indian sub-continent is very diverse in its climatic, biodiversity and agronomic landscape [13,14], which is likely to influence FAW population dynamics and its management. Indeed, the potential of native biological control agents, such as entomopathogenic fungi and parasitoids in FAW management has recently been demonstrated [15,16,17], but chemical control using synthetic insecticides remains most effective barring the potential selection for resistance and is recommended by the agricultural branch of the Indian government in the case of high FAW infestations [18].

The efficient control of FAW in the field can be challenging as later larval stages are less sensitive to insecticides and larvae lodge themselves inside the maize whorls, compromising the effect of insecticides acting by contact [19,20]. Moreover, FAW is currently among the top 15 most insecticide-resistant insect species [21] with cases reported to different chemical classes of insecticides, such as organophosphates, pyrethroids, spinosyns, diamides and benzoylureas [20,22,23,24,25], and *Bacillus thuringiensis* (Bt) insecticidal Cry proteins expressed in transgenic crops such as Cry1F, Cry1A.105 and Cry2Ab [26,27,28,29]. Increased detoxification and target-site mutations are the most common mechanisms conferring insecticide resistance in FAW [30,31,32]. In the case of high selection pressure in the field, more than one mechanism of resistance can be found in a single individual [30]. Moreover, one mechanism of resistance can confer resistance to a range of compounds, leading to cross-resistance [21]. Therefore, a study on the efficacy of different chemical classes of insecticides can provide information of important practical relevance for the implementation of an appropriate insecticide resistance management (IRM) strategy.

Target-site mutations conferring insecticide resistance and low field efficacy of older chemistries have been already reported from countries recently invaded [30,33], including India [34]. Therefore, it is important to establish an insecticide baseline susceptibility in FAW in India to make efficient decisions on what mode of action/chemical class to recommend for control [35]. Such baseline studies are inevitable to support practical decisions on application windows against FAW, composed of insecticides belonging to different modes of action and therefore, delaying resistance [21]. In a recent laboratory and field study in India, chlorantraniliprole, emamectin benzoate, and spinetoram showed satisfactory control of FAW populations collected from Southwest India [35]. Moreover, the use of chlorantraniliprole 18.5 SC, thiamethoxam 12.6% + lambda cyhalothrin 9.5% ZC, and spinetoram 1.7 SC are currently recommended in India as foliar applications by the Central Insecticide Board and Registration Committee to control FAW in maize [18].

Here, we conducted a large-scale baseline insecticide susceptibility monitoring with nine different insecticides against 47 FAW populations collected between 2019 and 2020 across India. Compounds with different modes of action representing acetylcholinesterase inhibitors, γ-aminobutyric acid (GABA)-gated chloride channel blockers, sodium channel modulators, nicotinic acetylcholine receptor allosteric modulators, glutamate-gated chloride channel allosteric modulators and ryanodine receptor modulators were tested (Table 1).

Moreover, we determined insecticide resistance ratios for FAW populations collected across India in comparison to an Indian population established in 2018 and a susceptible laboratory population collected in Brazil in 2005. The obtained data provide an important reference for future resistance monitoring initiatives and will help to detect early changes in insecticide efficacy to guide regional insecticide use and the implementation of IRM strategies.

## 2. Materials and Methods

### 2.1. Insect Origin and Rearing

Larvae of *S. frugiperda* were collected in the years 2018–2020 from maize fields at different sites in India (Supplementary Figure S1, Table S1) and reared on commercial artificial diet under controlled climate conditions (25 °C, 65% relative humidity and L16/D8 photoperiod) [36]. Larvae of the first generation (F1) were used in bioassays. Population I-2018 was collected in Bangalore, Karnataka in 2018, the year when *S. frugiperda* was first detected in India [4]; 24 FAW populations were collected in 2019 and 23 populations in 2020 (Supplementary Table S1). This population was maintained without insecticide selection pressure under the same conditions as described above and served as the Indian baseline population. A *S. frugiperda* population, collected in 2005 in Brazil named SUS-2005 was herein used as an insecticide susceptible reference population as described elsewhere [29,36].

### 2.2. Chemicals

Insecticides used in bioassays against the different populations were applied as commercial formulations (Table 1) diluted in tap water: thiodicarb (Larvin^®^ WP 75, Bayer CropScience, Maharashtra, India), chlorpyrifos (Lethal^®^ EC 20, Insecticides India Limited, Delhi, India), fipronil (Regent^®^ SC 5, Bayer CropScience, Maharashtra, India), deltamethrin (Decis^®^ EC 2.8, Bayer CropScience, Maharashtra, India), spinetoram (Delegate^®^ SC 11.7, Dow AgroSciences, Maharashtra, India), emamectin benzoate (Proclaim^®^ SG 5, Crystal Crop Protection, India), flubendiamide (Fame^®^ SC 480, Bayer CropScience, Maharashtra, Delhi, India), chlorantraniliprole (Coragen^®^ SC 18.5, FMC Corporate, Maharashtra, India), and tetraniliprole (Vayego^®^ SC 200, Bayer CropScience, Maharashtra, India). Fipronil is not registered for FAW control in India but was included as an alternative mode of action insecticide. For bioassays with population SUS-2005, the insecticides spinetoram (Agilent Technologies, Santa Clara, CA, USA), emamectin benzoate (Riedel-de-Haën, Sigma-Aldrich, Seelze, Germany), chlorpyrifos (Riedel-de-Haën, Sigma-Aldrich, Seelze, Germany) and thiodicarb (Riedel-de-Haën, Sigma-Aldrich, Seelze, Germany) were purchased as active ingredient standards of analytical grade. Emulsifier W (EW; CAS No. 104376–72-9) was used as a detergent and obtained from Lanxess (Lanxess, Leverkusen, Germany). Technical insecticides were dissolved in 0.01% (*v*/*v*) dimethylformamid-emulsifier W in aqueous solution at a concentration of 1000 µg/mL (spinetoram, emamectin-benzoate) or 1500 µg/mL (chlorpyrifos, thiodicarb) and further diluted to working solutions in double-distilled water.

### 2.3. Bioassays

Baseline susceptibility data were collected using a diet incorporation assay according to IRAC test method number 20 [37] with minor modifications. In brief, third instar larvae were individually placed onto 1 mL of artificial diet in each well of insect bioassay trays (Frontier Agriculture Sciences, Newark, DE, USA) mixed with serial dilutions of insecticides covering 0–100% mortality. Seven concentrations of each insecticide within the respective dose range stated in Table 1 were tested with distilled water serving as a negative control. Each replicate consisted of 16 larvae and the bioassay was repeated three times. The bioassays were incubated at 25 °C, 65% relative humidity and L16h/D8 photoperiod. Larval mortality was scored after five days. Data were not corrected for control mortality since control mortalities did not exceed 10%. Growth retarded larvae of approximately 1/3 in size of control larvae were considered strongly affected and therefore scored as dead.

### 2.4. Data Analysis

Mortality data were analyzed using Excel 365 (Microsoft Corporation, Redmond, WA, USA) and GraphPad Prism v9.0.2 (GraphPad Software, San Diego, CA, USA). Four parameter non-linear regression models (constraints: bottom = 0, top = 100) were applied to calculate EC_50_-and EC_95_-values and 95% confidence intervals. For the calculation of composite EC_50/95_-values, mortality data of all populations collected in 2019 or 2020 were combined and subjected to four parameter non-linear regression analyses. Resistance ratios (RR) were calculated by dividing the EC_50_-value of the respective field populations by the EC_50_-value of populations I-2018 and SUS-2005, respectively. For a direct comparison to EC_95_-values, recommended field doses of insecticides (Table 1) were converted to spray solution concentrations with an estimated average water usage of 500 L/ha.

## 3. Results

### 3.1. Insecticide Susceptibility of Indian FAW Populations

Third instar larvae of 47 different FAW populations collected across India in 2019 and 2020 were exposed to insecticides from different chemical classes for five days in diet incorporation bioassays to evaluate their efficacy. Some of the field-collected populations, particularly those sampled in 2019, could not be tested against all insecticides due to low numbers of F1 larvae. EC_50_ values for thiodicarb ranged from 2.91 to 16.0 µg/mL (Supplementary Table S2), chlorpyrifos 3.62–49.1 µg/mL (Supplementary Table S3), fipronil 3.77–15.8 µg/mL (Supplementary Table S4), deltamethrin 1.99–14.1 µg/mL (Supplementary Table S5), spinetoram 0.009–0.029 µg/mL (Supplementary Table S6), emamectin benzoate 0.003–0.058 µg/mL (Supplementary Table S7), flubendiamide 0.040–0.841 µg/mL (Supplementary Table S8), chlorantraniliprole 0.005–0.035 µg/mL (Supplementary Table S9), tetraniliprole 0.008–0.036 µg/mL (Supplementary Table S10).

**Figure 1:**
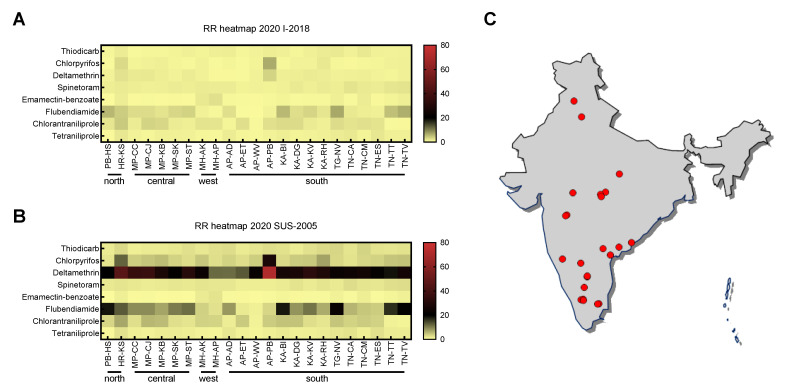
Comparative heatmaps of resistance ratios (RR) against eight insecticides of different populations of *Spodoptera frugiperda* collected in 2020 [30]. The RR was calculated based on EC_50_ values relative to (**A**) an Indian population I-2018 collected in the year of *S. frugiperda* invasion in India and (**B**) to a susceptible Brazilian population SUS-2005. (**C**) Map illustrating the 2020 sampling sites of 23 different Indian fall armyworm populations. Complete geographic details of all samples collected in 2019 and 2020 can be accessed in the supplementary information (Supplementary Table S1).

The EC_50_ values obtained for the field-collected populations were compared with the EC_50_ value of I-2018, a FAW population considered as the Indian reference, as it was collected at the time of the FAW invasion in 2018. The resulting resistance ratios (RR) for the populations collected in 2019 and 2020 were typically close to 1 in most populations, indicating similar insecticide sensitivity as population I-2018 across chemical classes, except for a few populations exhibiting higher RRs (Figure 1A, Supplementary Tables S2–S7 and S10). The EC_50_ values for flubendiamide (2019, 2020) and chlorantraniliprole (2020) were slightly, but significantly increased in several field-collected populations compared to I-2018, with maximum RRs of 7.36 and 6.84, respectively (Figure 1A, Supplementary Tables S8 and S9). 

However, the resistance status of invading FAW populations prior to settlement in new geographies—such as I-2018 in India—is usually unknown. Consequently, while comparison of field-collected FAW populations to I-2018 reveals general changes in insecticide resistance levels since FAW detection in India, it might still lead to misinterpretations of the actual insecticide resistance level. Therefore, we compared the EC_50_ values obtained for I-2018 with those of SUS-2005, an insecticide susceptible FAW reference population originally collected in Brazil. The differences in EC_50_ values between I-2018 and SUS-2005 were ≤2-fold for thiodicarb, fipronil, spinetoram, emamectin benzoate, chlorantraniliprole and tetraniliprole, suggesting similar susceptibility and lack of resistance. Whereas chlorpyrifos, flubendiamide and deltamethrin were significantly less effective against population I-2018 compared to SUS-2005 (Supplementary Tables S3, S5 and S8). The highest RR (21.6-fold) was observed for deltamethrin.

By utilizing SUS-2005 as a reference, high levels of deltamethrin resistance in all field-collected FAW populations collected in 2020 were found (Figure 1B,C, Supplementary Table S5). Population AP-PB originating from Andra Pradesh exhibited the greatest deltamethrin resistance level of 71.3-fold, followed by HR-KS from Haryana state with 45.9-fold resistance (Figure 1B, Supplementary Table S5). A significant increase in resistance levels by 3- and 4-fold could also be observed for flubendiamide and chlorpyrifos, respectively, resulting in RR as high as 24.5 for flubendiamide and 27.5 for chlorpyrifos (Figure 1B, Supplementary Tables S3 and S8). For thiodicarb, spinetoram, emamectin benzoate, and tetraniliprole, RR did not change when EC_50_ values of field populations were compared to population SUS-2005 (Figure 1B, Supplementary Tables S2, S6, S7 and S10), whereas slight changes were seen for chlorantraniliprole (Figure 1B, Supplementary Table S9). The data did not reveal a distinct pattern of insecticide resistance based on geography for those populations collected in 2020 (Figure 1B,C).

### 3.2. Temporal Development of Insecticide Resistance

**Figure 2:**
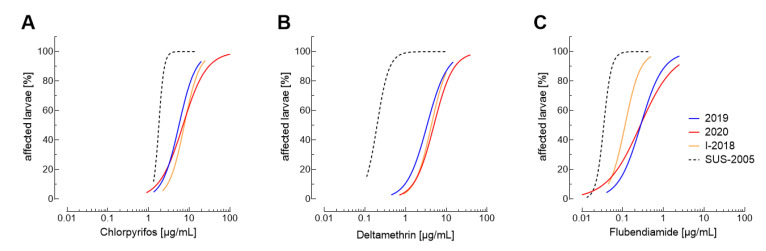
Composite log-dose probit-mortality data for *Spodoptera frugiperda* populations collected in 2019 (blue) and 2020 (red). Composite dose-response curves of combined bioassay data are shown for (**A**) chlorpyrifos, (**B**) deltamethrin and (**C**) flubendiamide. The dose-response curves obtained for the I-2018 population and the insecticide susceptible reference population SUS-2005 are shown in orange and black (dotted line), respectively. The plotted curves are based on data (including 95% confidence intervals) presented in Supplementary Table S11.

The quantification of resistance levels over the years helps to detect early changes in insecticide efficacy allowing adjustment of IRM strategies in affected regions. Here, we focused especially on chlorpyrifos, flubendiamide and deltamethrin, because particularly for these insecticides significant levels of resistance were observed (Figure 1). Composite 2019 and 2020 log-dose probit-mortality values revealed a clear distinction of the field-collected FAW populations from the susceptible reference population SUS-2005 for all three insecticides (Figure 2; Supplementary Table S11). For chlorpyriphos and deltamethrin hardly no differences in the overall efficacy were observed between populations collected in 2018, 2019 and 2020 (Figure 2A,B), whereas flubendiamide efficacy differed between the original population collected in 2018 and those collected in 2019 and 2020 (Figure 2C, Supplementary Table S11).

### 3.3. Comparison of Recommended Field Rates to EC_95_-Values for all Insecticides

**Figure 3:**
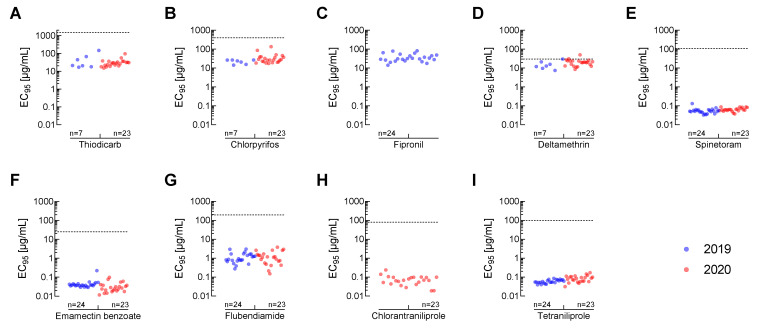
EC_95_-values (dots) for different insecticides against Indian *Spodoptera frugiperda* populations collected in 2019 (blue) and 2020 (red) treated with (**A**) thiodicarb, (**B**) chlorpyrifos, (**C**) fipronil, (**D**) deltamethrin, (**E**) spinetoram, (**F**) emamectin benzoate, (**G**) flubendiamide, (**H**) chlorantraniliprole and (**I**) tetraniliprole in comparison to recommended field application rates in India (dashed line). Insecticide spray concentrations were calculated based on information provided in Table 1 with an average water usage of 500 L/ha, except for fipronil which is not registered for FAW control in India. Number of populations (n) tested in the respective sampling years for each insecticide are indicated in each graph. The full data set including all calculated values can be accessed in the supplementary information (Supplementary Tables S2–S10).

To estimate the potential field efficacy of all tested chemical insecticides against FAW, we calculated and plotted the respective EC_95_-values for all field-collected populations along with the recommended label rates in India (Figure 3). For almost all compounds, EC_95_ values were considerably lower than the recommended field application rates irrespective of the collection year of the populations. The only exception is deltamethrin, where the EC_95_-value was close to or in the case of population AP-PB above the recommended label rate (Figure 3D). For fipronil, no label rate was provided because this compound is not recommended for use against FAW in India (Figure 3C). The lowest variation in EC_95_-values across sampling sites was observed for tetraniliprole and spinetoram (Figure 3E,I).

## 4. Discussion

After its first detection in Karnataka state in May 2018 [4,17,38], FAW spread to almost all states in India by August 2019 [39,40]. To provide the basis for sound recommendations to control this new invasive lepidopteran pest species, we conducted a baseline susceptibility study for the Indian sub-continent covering nine different insecticides out of six MoA classes using 47 FAW populations collected in 2019 and 2020.

Low resistance ratios were observed for thiodicarb, spinetoram, emamectin benzoate, chlorantraniliprole and tetraniliprole across field-samples of both collection years when compared to a laboratory susceptible reference population SUS-2005. Our results correspond with Indian FAW laboratory (leaf-dip) and field experiments conducted in 2018, and which previously demonstrated high efficacy of spinetoram, emamectin benzoate and chlorantraniliprole [35]. In contrast, chlorpyrifos, deltamethrin and flubendiamide were less effective against field populations collected in 2019 and 2020 in comparison to the reference population SUS-2005 (Figure 1, Supplementary Tables S3, S5 and S8). Likewise, population I-2018 (collected in the year of FAW invasion) did also show low-to-moderate levels of resistance to these insecticides when compared to SUS-2005. A finding underpinning the importance of utilizing established susceptible reference populations to assess the extent of the resistance levels present at the time of invasion.

Genomic analyses of a FAW population collected in 2018 from Karnataka state in India detected the presence of the F290V target-site mutation in the acetylcholinesterase (AChE), along with the less prevalent A201S mutation [34]. The F290V substitution is known to confer resistance to organophosphates in FAW [20], but also cross-resistance to carbamates has been described, e.g., against carbaryl in *Cydia pomonella* [41]. The high frequency of target-site mutations in AChE in the invasive FAW population in India might support the lower relative RRs when EC_50_ values of the field populations were compared to I-2018 rather than SUS-2005, because SUS-2005 has been characterized earlier and is known to lack the F290V mutation in AChE [42]. However, we did not find a correlation between the RR’s calculated for chlorpyrifos and the carbamate thiodicarb (Pearson’s r 0.24; Tables S2 and S3). More investigations at the biochemical level are required to understand and explain potential cross-resistance issues between organophosphates and carbamates in Indian FAW populations.

Based on our findings we can conclude that moderate levels of deltamethrin resistance were already present in FAW populations invading India in 2018. However, this fact only became obvious after a comparison of the efficacy data obtained for the Indian field-collected populations to the susceptible reference SUS-2005 (Figure 2). A recent mechanistic investigation revealed that the resistance to pyrethroids in FAW might be metabolic and based on the increased expression and activity of cytochrome P450 enzymes, particularly those belonging to the CYP9A subfamily [42]. Interestingly, CYP9A enzymes were recently shown to detoxify pyrethroid insecticides—including deltamethrin—in the cotton bollworm *Helicoverpa armigera* [43]. An increased metabolic turnover could explain the relatively high RR values obtained for deltamethrin in our study. Nevertheless, it is still unknown for FAW whether this mechanism would confer cross-resistance to all pyrethroids at similar or different levels. However, a previous study on a field-collected Brazilian population selected for pyrethroid resistance, three voltage-gated sodium channel mutations, T929I, L932F and L1014F, were detected—albeit at very low frequency despite continuous selection pressure, thus reinforcing the importance of metabolic pyrethroid resistance in FAW [20,42]. Another study on FAW samples collected outside Brazil revealed that only a single population from Indonesia carried a target-site mutation (L1014F) in the voltage-gated sodium channel among populations from different invaded countries analyzed [30,32,34].

For flubendiamide, population I-2018 exhibited low levels of resistance compared to the Brazilian susceptible population SUS-2005. Significant differences between the EC_50/95_-value of population I-2018 and calculated composite EC_50_- and EC_95_-values of 2019 and 2020 FAW field populations indicated additional selection pressure that further enhanced flubendiamide resistance levels. High levels of cross-resistance among diamide insecticides (including flubendiamide) in FAW have been recently described in a population from Brazil after laboratory selection with chlorantraniliprole [22] and was shown to be mediated by a mutation (I4790M) in the ryanodine receptor, the diamide target-site [44]. The same mutation has been shown to confer diamide resistance in a few other lepidopteran pests such as diamondback moth, beet armyworm and tomato leafminer [45]. However, the frequency of this resistance allele in FAW field populations seems very low, as it was not yet detected in populations collected outside Brazil [32,34,44], except China where it was detected at very low frequency in a FAW population showing low levels of diamide resistance [46]. However, it remains elusive and needs to be analyzed if the moderate RR obtained for flubendiamide in our study is due to an increased detoxification capacity or the presence of a ryanodine receptor target-site mutation, or both. The lack of any cross-resistance to tetraniliprole and almost no cross-resistance to chlorantraniliprole might indicate the absence of a ryanodine receptor target-site mutation affecting diamide binding in Indian FAW populations. As it is generally known that the presence of such a mutation confers cross-resistance among diamide chemotypes [45].

The absence of tetraniliprole cross-resistance renders it a valuable tool in future resistance management strategies. Nevertheless, an adequate IRM strategy should consider the rotation of diamide applications with insecticides belonging to other modes of action in order to increase the life span of diamides in the market. Future resistance monitoring efforts should include all chemical classes of insecticides available for FAW control in India. This is a laborious task that could be simplified by establishing discriminating dose bioassays for the individual insecticides, e.g., based on the EC_95_-values (or slightly higher concentrations) against a susceptible reference population such as SUS-2005 used in this study. This would allow the detection of early signs of resistance development.

Our study demonstrated resistance to some chemical classes of insecticides such as organophosphates and pyrethroids in Indian FAW, most likely already present in populations that invaded Karnataka in 2018. The initial populations from Karnataka state were shown to be closely related to South African populations of corn and rice strain haplotype hybrids according to original host preference classification in the Americas [9,47]. Since then, FAW has rapidly adapted to the Indian landscape despite the different agroclimatic zones and experienced an expansion mediated by its population dynamics [9]. This is in line with our findings of a generally homogenous distribution of resistance with only a few geographically unlinked hotspots.

## 5. Conclusions

Our study described a robust and reliable bioassay format and provided a useful baseline for future FAW resistance monitoring initiatives in India. The presence of resistance alleles in Indian FAW populations conferring low-to-moderate levels of resistance to some chemical classes of insecticides highlights the need for continued monitoring efforts to limit the dispersal of resistant haplotypes. Furthermore, this will facilitate the implementation of appropriate IRM strategies at regional and local levels.

## Figures and Tables

**Table 1:** Insecticide formulations tested against fall armyworm, including their respective mode of action according to the IRAC (Insecticide Resistance Action Committee) classification scheme, and recommended dose rates for *Spodoptera frugiperda* control in India.

IRAC MoA Class	Chemical Class	MoA ^1^	Active Ingredient	Formulation	Recommended Dose [g a.i./ha]	Dose Range in Bioassays [µg/mL]
1A	Carbamates	AChE ^2^ inhibitor	Thiodicarb	75% WP ^7^	750	0.7–40
1B	Organophos-phates	AChE ^2^ inhibitor	Chlorpyrifos	20% EC ^8^	200	0.87–100
2B	Fiproles	GABA ^3^-gated chloride channel blockers	Fipronil	5% SC ^9^	not registered	0.3–30
3A	Pyrethroids	Sodium channel modulator	Deltamethrin	2.8% EC ^8^	15	0.11–40
5	Spinosyns	nAChR ^4^ allosteric modulator	Spinetoram	11.7% SC ^9^	54	0.001–0.09
6	Avermectins	GluCl ^5^ allosteric modulator	Emamectin benzoate	5% SG ^10^	12.5	0.001–0.15
28	Diamides	RyR ^6^ modulator	Flubendiamide	480 SC ^9^	96	0.009–2.5
			Chlorantra-niliprole	18.5% SC ^9^	40	0.001–0.2
			Tetraniliprole ^11^	200 SC ^9^	50	0.002–0.07

^1^ MoA: mode of action; ^2^ AChE: acetylcholinesterase; ^3^ GABA: γ-aminobutyric acid; ^4^ nAChR: nicotinic acetylcholine receptor; ^5^ GluCl: glutamate-gated chloride channel; ^6^ RyR: ryanodine receptor; ^7^ WP: wettable powder; ^8^ EC: emulsifiable concentrate; ^9^ SC: suspension concentrate; ^10^ SG: water-soluble granule; ^11^ Not yet officially labelled for FAW control.

## Data Availability

We will provide all data generated in this study upon request.

## Supplementary Materials

The following are available online at https://www.mdpi.com/2075-4450/12/8/758/s1, Figure S1: schematic map of FAW collection sites, Table S1: FAW sampling details, Table S2: Log-dose mortality data for thiodicarb, Table S3: Log-dose mortality data for chlorpyrifos, Table S4: Log-dose mortality data for fipronil, Table S5: Log-dose mortality data for deltamethrin, Table S6: Log-dose mortality data for spinetoram, Table S7: Log-dose mortality data for emamectin benzoate, Table S8: Log-dose mortality data for flubendiamide, Table S9: Log-dose mortality data for chlorantraniliprole, Table S10: Log-dose mortality data for tetraniliprole, Table S11: composite mortality data.
