# Supplementary material for: Baseline Susceptibility of Spodoptera frugiperda Populations Collected in India towards Different Chemical Classes of Insecticides
Source: Insects. 2021 Aug 23;12(8):758. doi: 10.3390/insects12080758 (PMC8397139; doi:10.3390/insects12080758)
Supplement: Supplementary file 1 [file insects-12-00758-s001.zip › insects-1291806-supplementary.pdf]

insects
MDPI
Supplementary Information

Baseline susceptibility of *Spodoptera frugiperda* populations collected in India towards different chemical classes of insecticides

Mahesh Kulye [1], Sonja Mehlhorn [2], Debora Boaventura [2], Nigel Godley [2], Sreedevi K V [1], Thimmaraju Rudrappa [1], Tara Charan [3], Dinesh Rathi [3], and Ralf Nauen [2,*]

[1] Bayer AG, Crop Science Division, Bangalore, 560045, India; mahesh.kulye@bayer.com; sreedevi.kv@bayer.com; thimmaraju.rudrappa@bayer.com
[2] Bayer AG, Crop Science Division, R&D, Alfred Nobel Str. 50, 40789 Monheim, Germany; sonja.mehlhorn@bayer.com; debora.duarteboaventura@bayer.com; nigel.godley@bayer.com; ralf.nauen@bayer.com
[3] Bayer AG, CoWrks India Private, New Delhi, 110037, India; tara.chan@bayer.com; dinesh.rathi@bayer.com

* Correspondence: *ralf.nauen@bayer.com*

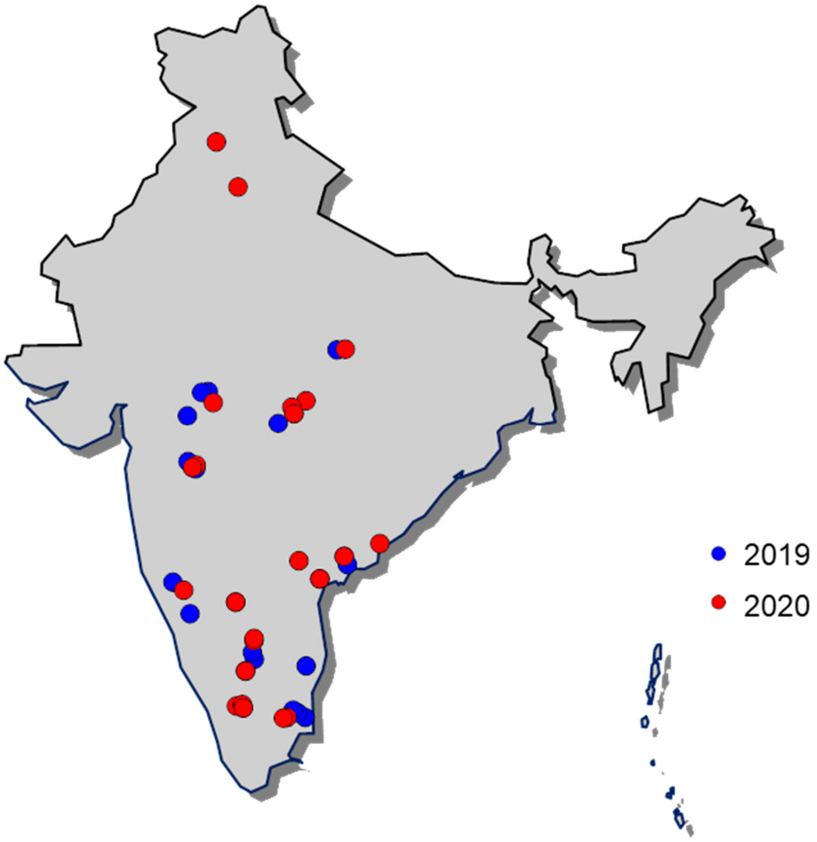

Figure S1. Schematic map of *Spodoptera frugiperda* collection sites across India in 2019 (blue) and 2020 (red). The schematic map was created using EasyMap software (Lutum+Tappert DV-Beratung GmbH, Bonn, Germany).

Table S1. Details on *Spodoptera frugiperda* sampling sites and years

Region	Sample ID	State	District	City	Year
South	SUS-I	Karnataka	Bangalore Rural	Doddaballapura	2018
Central	MP-BB	Madhya Pradesh	Barwani	Badgaon	2019
Central	MP-CC	Madhya Pradesh	Chhindwara	Chand	2019
Central	MP-CCD	Madhya Pradesh	Chhindwara	Chhindwara	2019
Central	MP-IA	Madhya Pradesh	Indore	Aurangapura	2019
Central	MP-ID	Madhya Pradesh	Indore	Dhannad	2019
Central	MP-SM	Madhya Pradesh	Satna	Maihar	2019
West	MH-AG	Maharashtra	Aurangabad	Ganori	2019
West	MH-AH	Maharashtra	Aurangabad	Hatnur	2019
West	MH-AK	Maharashtra	Aurangabad	Khultabad	2019
South	AP-KG	Andhra Pradesh	Krishna	Gannavaram	2019
South	AP-PB	Andhra Pradesh	Prakasam	Bobbepalli	2019
South	AP-WV	Andhra Pradesh	West Godhavari	Vijayarai	2019
South	KA-BA	Karnataka	Bangalore Rural	Aralu mallige	2019
South	KA-BS	Karnataka	Bangalore Rural	Shampur	2019
South	KA-BI	Karnataka	Bellary	Ibrahimpura	2019
South	KA-BN	Karnataka	Belgaum	Narendra Nagar	2019
South	KA-HA	Karnataka	Haveri	Akkialur	2019
South	KA-RH	Karnataka	Ramanagara	Haniyur	2019
South	TN-AR	Tamil Nadu	Ariyalur	Reddipalayam	2019
South	TN-AS1	Tamil Nadu	Ariyalur	Sripuranthan	2019
South	TN-AS2	Tamil Nadu	Ariyalur	Sripuranthan	2019
South	TN-CD	Tamil Nadu	Coimbatore	Dasapalayam	2019
South	TN-TA	Tamil Nadu	Tiruvarur	Allikulam	2019
South	TN-TP	Tamil Nadu	Tiruvannamalai	Pudupalayam	2019
North	PB-HS	Punjab	Hoshiapur	Simbli	2020
North	HR-KS	Haryana	Karnal	Sohana	2020
Central	MP-CC	Madhya Pradesh	Chhindwara	Chand	2020
Central	MP-CJ	Madhya Pradesh	Chhindwara	Jhirlinga	2020
Central	MP-KB	Madhya Pradesh	Khargone	Barwaha	2020
Central	MP-SK	Madhya Pradesh	Satna	Kandwari	2020
Central	MP-ST	Madhya Pradesh	Seoni	Thaori	2020
West	MH-AK	Maharashtra	Aurangabad	Kultabad	2020
West	MH-AP	Maharashtra	Aurangabad	Phulmbri	2020
South	AP-AD	Andra Pradesh	Anantapur	Demakethapalli	2020
South	AP-ET	Andra Pradesh	East Godhavari	Thimmapuram	2020
South	AP-WV	Andra Pradesh	West Godhavari	Vijayarai	2020
South	AP-PB	Andra Pradesh	Prakasam	Bobbepalli	2020
South	KA-BI	Karnataka	Bellary	Ibrahimpura	2020
South	KA-DG	Karnataka	Dharwad	Garag	2020
South	KA-KV	Karnataka	Kolar	Vatadahosahalli	2020
South	KA-RH	Karnataka	Ramanagara	Haniyur	2020
South	TG-NV	Telangana	Nalgonda	Vijayapuri North	2020
South	TN-CA	Tamil Nadu	Coimbature	Allapalayam	2020
South	TN-CM	Tamil Nadu	Coimbature	Meenampalayam	2020
South	TN-ES	Tamil Nadu	Erode	Salai palayam	2020
South	TN-TT	Tamil Nadu	Tiruchirappalli	Athigudi	2020
South	TN-TV	Tamil Nadu	Tiruchirappalli	Varakuppai	2020

Table S2. Log-dose mortality data for thiodicarb against third instar larvae of Indian populations of *Spodoptera frugiperda* in diet incorporation assays based on IRAC method no. 20. Mortality was assessed five days after treatment.

Sample ID	Year	EC_{50} [μg/mL]	95% CI[1]	Slope (±SE[2])	RR[3] to SUS-2005	RR[3] to I-2018	EC_{95} [μg/mL]	95% CI[1]
SUS-2005	2005	3.566	3.424 - 3.715	5.16 (0.43)	1.00	0.51	6.312	5.668 - 7.030
I-2018	2018	7.007	6.540 - 7.508	2.04 (0.13)	1.96	1.00	29.580	24.140 - 36.250
MP-CC	2019	4.473	4.132 - 4.841	1.90 (0.13)	1.25	0.64	21.040	16.580 - 26.700
MP-SM	2019	6.437	6.043 - 6.858	1.53 (0.07)	1.81	0.92	44.350	35.980 - 54.660
MH-AG	2019	2.912	2.579 - 3.288	1.68 (0.17)	0.82	0.42	16.860	11.400 - 24.940
AP-KG	2019	4.488	4.159 - 4.843	1.95 (0.13)	1.26	0.64	20.380	16.230 - 25.580
KA-BA	2019	13.280	12.470 - 14.150	1.82 (0.10)	3.72	1.90	66.840	54.490 - 81.980
KA-HA	2019	4.460	4.194 - 4.743	2.11 (0.13)	1.25	0.64	17.960	14.860 - 21.720
TN-TA	2019	16.020	14.620 - 17.550	1.32 (0.09)	4.49	2.29	147.900	103.900 - 210.800
PB-HS	2020	5.375	5.089 - 5.676	2.45 (0.14)	1.51	0.77	17.850	15.260 - 20.880
HR-KS	2020	10.100	9.512 - 10.710	2.22 (0.13)	2.83	1.44	38.080	31.870 - 45.490
MP-CC	2020	4.285	3.998 - 4.593	2.33 (0.17)	1.20	0.61	15.130	12.400 - 18.470
MP-CJ	2020	5.332	4.907 - 5.792	2.11 (0.17)	1.50	0.76	21.570	16.860 - 27.590
MP-KB	2020	5.491	5.192 - 5.806	2.54 (0.16)	1.54	0.78	17.490	14.930 - 20.490
MP-SK	2020	6.260	5.948 - 6.588	2.23 (0.11)	1.76	0.89	23.450	20.220 - 27.210
MP-ST	2020	8.154	7.563 - 8.792	1.97 (0.13)	2.29	1.16	36.390	29.030 - 45.610
MH-AK	2020	6.172	5.953 - 6.399	2.63 (0.11)	1.73	0.88	18.890	17.060 - 20.920
MH-AP	2020	8.289	7.888 - 8.710	2.60 (0.14)	2.32	1.18	25.740	22.370 - 29.620
AP-AD	2020	9.329	8.744 - 9.953	2.53 (0.18)	2.62	1.33	29.840	24.830 - 35.880
AP-ET	2020	5.493	4.950 - 6.096	1.74 (0.15)	1.54	0.78	29.720	21.360 - 41.370
AP-WV	2020	6.982	6.629 - 7.354	2.53 (0.14)	1.96	1.00	22.350	19.270 - 25.930
AP-PB	2020	9.061	8.785 - 9.345	2.63 (0.09)	2.54	1.29	27.780	25.450 - 30.330
KA-BI	2020	8.101	7.649 - 8.580	2.10 (0.11)	2.27	1.16	32.940	27.850 - 38.970
KA-DG	2020	5.141	4.867 - 5.430	2.15 (0.11)	1.44	0.73	20.250	17.260 - 23.740
KA-KV	2020	7.993	7.498 - 8.521	2.50 (0.17)	2.24	1.14	25.910	21.610 - 31.080
KA-RH	2020	10.260	9.564 - 11.010	1.73 (0.10)	2.88	1.46	56.210	45.020 - 70.180
TG-NV	2020	11.290	10.67 - 11.950	2.53 (0.16)	3.17	1.61	36.170	30.720 - 42.580
TN-CA	2020	7.213	6.633 - 7.843	1.82 (0.13)	2.02	1.03	36.400	28.200 - 46.980
TN-CM	2020	11.760	11.000 - 12.560	1.41 (0.07)	3.30	1.68	95.220	74.760 - 121.300
TN-ES	2020	7.424	6.958 - 7.921	2.07 (0.12)	2.08	1.06	30.850	25.490 - 37.330
TN-TT	2020	6.784	6.320 - 7.282	1.92 (0.12)	1.90	0.97	31.550	25.530 - 39.000
TN-TV	2020	7.606	7.193 - 8.043	2.11 (0.11)	2.13	1.09	30.790	26.130 - 36.270

[1]CI: confidence interval; [2]SE: standard error; [3]RR: resistance ratio, EC_{50} values of respective population divided by EC_{50} of population SUS-2005 or I-2018

Table S3. Log-dose mortality data for chlorpyrifos against third instar larvae of Indian populations of *Spodoptera frugiperda* in diet incorporation assays based on IRAC method no. 20. Mortality was assessed five days after treatment.

Sample ID	Year	EC_{50} [µg/mL]	95% CI[1]	Slope (±SE[2])	RR[3] to SUS-2005	RR[3] to I-2018	EC_{95} [µg/mL]	95% CI[1]
SUS-2005	2005	1.783	1.693 - 1.879	6.72 (0.95)	1.00	0.24	2.763	2.419 - 3.157
I-2018	2018	7.509	7.119 - 7.921	2.31 (0.12)	4.21	1.00	26.870	23.040 - 31.330
MP-CC	2019	6.341	5.773 - 6.965	2.07 (0.19)	3.56	0.84	26.250	19.680 - 35.000
MP-SM	2019	5.866	5.438 - 6.328	1.94 (0.13)	3.29	0.78	26.710	21.300 - 33.500
MH-AG	2019	4.025	3.763 - 4.306	2.34 (0.16)	2.26	0.54	14.210	11.710 - 17.240
AP-KG	2019	5.534	5.173 - 5.919	2.02 (0.12)	3.10	0.74	23.790	19.470 - 29.050
KA-BA	2019	6.162	5.759 - 6.593	2.43 (0.18)	3.46	0.82	20.680	16.960 - 25.200
KA-HA	2019	3.964	3.628 - 4.331	2.15 (0.18)	2.22	0.53	15.560	12.020 - 20.130
TN-TA	2019	8.207	7.701 - 8.746	2.49 (0.18)	4.60	1.09	26.820	22.200 - 32.390
PB-HS	2020	3.620	3.410 - 3.843	1.81 (0.09)	2.03	0.48	18.460	15.310 - 22.250
HR-KS	2020	21.700	20.510 - 22.950	2.13 (0.11)	12.17	2.89	86.290	73.280 - 101.600
MP-CC	2020	6.730	6.314 - 7.174	2.02 (0.12)	3.77	0.90	28.890	23.920 - 34.900
MP-CJ	2020	8.024	7.427 - 8.670	1.88 (0.13)	4.50	1.07	38.560	30.470 - 48.780
MP-KB	2020	9.223	8.621 - 9.866	2.02 (0.12)	5.17	1.23	39.660	32.470 - 48.450
MP-SK	2020	6.077	5.775 - 6.394	2.22 (0.11)	3.41	0.81	22.850	19.710 - 26.480
MP-ST	2020	4.731	4.382 - 5.107	1.93 (0.13)	2.65	0.63	21.820	17.320 - 27.490
MH-AK	2020	7.724	7.099 - 8.404	2.05 (0.16)	4.33	1.03	32.560	25.370 - 41.790
MH-AP	2020	4.323	4.040 - 4.625	2.04 (0.13)	2.42	0.58	18.310	15.000 - 22.350
AP-AD	2020	6.021	5.656 - 6.411	2.01 (0.11)	3.38	0.80	26.120	21.660 - 31.510
AP-ET	2020	4.601	4.373 - 4.840	2.21 (0.11)	2.58	0.61	17.410	15.040 - 20.160
AP-WV	2020	6.831	6.421 - 7.268	2.18 (0.13)	3.83	0.91	26.370	21.930 - 31.710
AP-PB	2020	49.100	45.880 - 52.560	2.90 (0.25)	27.54	6.54	135.500	111.100 - 165.200
KA-BI	2020	5.144	4.619 - 5.728	2.24 (0.19)	2.89	0.69	19.190	15.880 - 23.190
KA-DG	2020	7.249	6.827 - 7.698	2.26 (0.14)	4.07	0.97	26.710	22.400 - 31.850
KA-KV	2020	7.418	7.046 - 7.810	1.88 (0.09)	4.16	0.99	35.570	30.030 - 42.140
KA-RH	2020	12.790	11.920 - 13.730	2.14 (0.15)	7.17	1.70	50.560	41.060 - 62.260
TG-NV	2020	5.411	5.030 - 5.820	2.25 (0.16)	3.03	0.72	20.020	16.170 - 24.780
TN-CA	2020	3.812	3.593 - 4.045	1.71 (0.08)	2.14	0.51	21.310	17.600 - 25.790
TN-CM	2020	5.701	5.265 - 6.172	1.95 (0.14)	3.20	0.76	25.720	20.260 - 32.640
TN-ES	2020	6.115	5.718 - 6.541	1.93 (0.11)	3.43	0.81	28.210	23.010 - 34.600
TN-TT	2020	8.036	7.655 - 8.436	1.70 (0.07)	4.51	1.07	45.430	38.640 - 53.420
TN-TV	2020	8.493	7.876 - 9.159	2.00 (0.14)	4.76	1.13	37.150	29.620 - 46.600

[1]CI: confidence interval; [2]SE: standard error; [3]RR: resistance ratio, EC_{50} values of respective population divided by EC_{50} of population SUS-2005 or I-2018

Table S4. Log-dose mortality data for fipronil against third instar larvae of Indian populations of *Spodoptera frugiperda* in diet incorporation assays based on IRAC method no. 20. Mortality was assessed five days after treatment.

Sample ID	Year	EC_{50} [μg/mL]	95% CI[1]	Slope (±SE[2])	RR[3] to SUS-2005	RR[3] to I-2018	EC_{95} [μg/mL]	95% CI[1]
SUS-2005	2005	6.549	6.169 - 6.952	4.91 (0.64)	1.00	1.44	11.930	10.030 - 14.180
I-2018	2018	4.558	4.358 - 4.767	2.61 (0.13)	0.70	1.00	14.110	12.430 - 16.020
MP-BB	2019	7.919	7.485 - 8.378	2.27 (0.13)	1.21	1.74	29.050	24.690 - 34.170
MP-CC	2019	10.450	9.489 - 11.510	1.62 (0.13)	1.60	2.29	64.560	45.940 - 90.710
MP-CCD	2019	5.768	5.421 - 6.138	1.94 (0.11)	0.88	1.27	26.340	21.880 - 31.700
MP-IA	2019	3.774	3.547 - 4.014	2.24 (0.14)	0.58	0.83	14.070	11.760 - 16.840
MP-ID	2019	5.788	5.541 - 6.046	2.37 (0.11)	0.88	1.27	20.040	17.690 - 22.700
MP-SM	2019	15.820	14.560 - 17.190	1.84 (0.14)	2.42	3.47	78.590	59.430 - 103.900
MH-AG	2019	8.875	8.538 - 9.225	3.06 (0.15)	1.36	1.95	23.260	20.880 - 25.930
MH-AH	2019	6.322	5.852 - 6.830	1.86 (0.13)	0.97	1.39	30.700	24.020 - 39.240
MH-AK	2019	8.803	8.245 - 9.399	1.62 (0.09)	1.34	1.93	54.440	43.600 - 67.980
AP-KG	2019	8.995	8.447 - 9.579	2.54 (0.17)	1.37	1.97	28.680	23.990 - 34.280
AP-PB	2019	5.997	5.642 - 6.375	2.13 (0.12)	0.92	1.32	23.860	19.960 - 28.520
AP-WV	2019	5.735	5.193 - 6.332	1.72 (0.14)	0.88	1.26	31.910	23.450 - 43.430
KA-BA	2019	9.073	7.956 - 10.350	1.75 (0.19)	1.39	1.99	48.790	32.420 - 73.420
KA-BS	2019	4.502	3.865 - 5.245	1.3 (0.12)	0.69	0.99	43.630	27.560 - 69.070
KA-BI	2019	9.549	8.921 - 10.220	2.39 (0.17)	1.46	2.09	32.810	26.990 - 39.890
KA-BN	2019	10.160	9.554 - 10.810	1.64 (0.08)	1.55	2.23	61.060	50.080 - 74.450
KA-HA	2019	14.590	13.280 - 16.040	1.71 (0.14)	2.23	3.20	81.410	59.100 - 112.100
KA-RH	2019	4.645	4.147 - 5.204	1.56 (0.12)	0.71	1.02	30.510	21.960 - 42.390
TN-AR	2019	5.560	5.171 - 5.978	2.16 (0.15)	0.85	1.22	21.770	17.560 - 27.000
TN-AS1	2019	9.909	9.392 - 10.450	2.21 (0.12)	1.51	2.17	37.590	32.150 - 43.960
TN-AS2	2019	4.516	4.197 - 4.859	2.16 (0.15)	0.69	0.99	17.660	14.260 - 21.860
TN-CD	2019	7.837	7.212 - 8.518	2.02 (0.16)	1.20	1.72	33.580	26.050 - 43.270
TN-TA	2019	7.853	7.198 - 8.568	1.7 (0.12)	1.20	1.72	44.460	33.810 - 58.480
TN-TP	2019	6.923	6.601 - 7.262	2.12 (0.1)	1.06	1.52	27.790	24.130 - 32.000

[1]CI: confidence interval; [2]SE: standard error; [3]RR: resistance ratio, EC_{50} values of respective population divided by EC_{50} of population SUS-2005 or I-2018

Table S5. Log-dose mortality data for deltamethrin against third instar larvae of Indian populations of *Spodoptera frugiperda* in diet incorporation assays based on IRAC method no. 20. Mortality was assessed five days after treatment.

Sample ID	Year	EC_{50} [μg/mL]	95% CI[1]	Slope (±SE[2])	RR[3] to SUS-2005	RR[3] to I-2018	EC_{95} [μg/mL]	95% CI[1]
SUS-2005	2005	0.198	0.190 - 0.206	2.94 (0.16)	1.00	0.05	0.538	0.481 - 0.602
I-2018	2018	4.271	3.946 - 4.623	2.12 (0.16)	21.61	1.00	17.080	13.420 - 21.750
MP-CC	2019	3.177	2.934 - 3.441	2.22 (0.18)	16.08	0.74	11.950	9.424 - 15.140
MP-SM	2019	4.802	4.437 - 5.196	1.99 (0.14)	24.30	1.12	21.150	16.710 - 26.770
MH-AG	2019	2.422	2.226 - 2.636	2.14 (0.17)	12.26	0.57	9.617	7.516 - 12.300
AP-KG	2019	2.450	2.260 - 2.656	1.78 (0.12)	12.40	0.57	12.800	9.980 - 16.410
KA-BA	2019	4.240	3.944 - 4.558	2.24 (0.16)	21.46	0.99	15.820	12.830 - 19.500
KA-HA	2019	1.985	1.834 - 2.148	2.23 (0.18)	10.05	0.46	7.441	5.878 - 9.421
TN-TA	2019	6.404	5.769 - 7.108	1.92 (0.18)	32.41	1.50	29.660	21.350 - 41.210
PB-HS	2020	4.055	3.860 - 4.260	2.53 (0.14)	20.52	0.95	12.990	11.300 - 14.940
HR-KS	2020	9.070	8.561 - 9.609	2.72 (0.19)	45.90	2.12	26.780	22.630 - 31.690
MP-CC	2020	7.022	6.624 - 7.445	2.38 (0.15)	35.54	1.64	24.180	20.420 - 28.620
MP-CJ	2020	7.180	6.779 - 7.604	2.03 (0.11)	36.34	1.68	30.700	25.910 - 36.370
MP-KB	2020	5.529	5.175 - 5.908	2.04 (0.13)	27.98	1.29	23.380	19.150 - 28.540
MP-SK	2020	4.160	3.959 - 4.370	2.19 (0.11)	21.05	0.97	15.940	13.820 - 18.400
MP-ST	2020	6.174	5.825 - 6.544	2.18 (0.12)	31.24	1.45	23.910	20.180 - 28.320
MH-AK	2020	4.563	4.388 - 4.745	2.90 (0.14)	23.09	1.07	12.570	11.270 - 14.030
MH-AP	2020	2.313	2.203 - 2.428	2.26 (0.11)	11.71	0.54	8.537	7.416 - 9.827
AP-AD	2020	2.264	2.118 - 2.420	1.86 (0.11)	11.46	0.53	10.990	8.991 - 13.440
AP-ET	2020	2.562	2.354 - 2.790	1.93 (0.15)	12.97	0.60	11.780	9.124 - 15.220
AP-WV	2020	4.384	4.173 - 4.605	1.75 (0.08)	22.19	1.03	23.590	19.880 - 28.000
AP-PB	2020	14.090	13.250 - 14.990	2.34 (0.15)	71.31	3.30	49.580	41.420 - 59.340
KA-BI	2020	5.183	4.833 - 5.557	2.19 (0.15)	26.23	1.21	19.880	16.200 - 24.40
KA-DG	2020	5.391	5.089 - 5.712	2.30 (0.13)	27.28	1.26	19.380	16.380 - 22.930
KA-KV	2020	6.401	6.170 - 6.641	2.53 (0.10)	32.39	1.50	20.510	18.470 - 22.760
KA-RH	2020	5.741	5.391 - 6.113	2.32 (0.15)	29.05	1.34	20.460	16.970 - 24.670
TG-NV	2020	4.468	4.194 - 4.760	2.00 (0.11)	22.61	1.05	19.450	16.120 - 23.470
TN-CA	2020	4.781	4.460 - 5.125	2.01 (0.13)	24.20	1.12	20.630	16.780 - 25.370
TN-CM	2020	5.052	4.599 - 5.549	1.80 (0.14)	25.57	1.18	25.880	19.350 - 34.630
TN-ES	2020	4.190	4.002 - 4.387	2.23 (0.10)	21.20	0.98	15.710	13.760 - 17.940
TN-TT	2020	3.663	3.510 - 3.824	2.41 (0.11)	18.54	0.86	12.460	11.020 - 14.090
TN-TV	2020	4.998	4.679 - 5.339	2.01 (0.12)	25.29	1.17	21.640	17.760 - 26.350

[1]CI: confidence interval; [2]SE: standard error; [3]RR: resistance ratio, EC_{50} values of respective population divided by EC_{50} of population SUS-2005 or I-2018

Table S6. Log-dose mortality data for spinetoram against third instar larvae of Indian populations of *Spodoptera frugiperda* in diet incorporation assays based on IRAC method no. 20. Mortality was assessed five days after treatment.

Sample ID	Year	EC_{50} [µg/mL]	95% CI[1]	Slope (±SE[2])	RR[3] to SUS-2005	RR[3] to I-2018	EC_{95} [µg/mL]	95% CI[1]
SUS-2005	2005	0.010	0.009 - 0.012	2.35 (0.37)	1.00	0.78	0.036	0.024 - 0.055
I-2018	2018	0.013	0.012 - 0.014	2.25 (0.13)	1.28	1.00	0.049	0.041 - 0.058
MP-BB	2019	0.021	0.020 - 0.023	3.29 (0.26)	2.07	1.62	0.052	0.045 - 0.061
MP-CC	2019	0.015	0.014 - 0.016	2.46 (0.14)	1.45	1.13	0.049	0.043 - 0.057
MP-CCD	2019	0.029	0.027 - 0.030	1.92 (0.08)	2.78	2.17	0.133	0.113 - 0.155
MP-IA	2019	0.023	0.021 - 0.024	3.16 (0.24)	2.21	1.73	0.058	0.049 - 0.067
MP-ID	2019	0.016	0.015 - 0.017	2.50 (0.14)	1.54	1.21	0.051	0.044 - 0.060
MP-SM	2019	0.022	0.021 - 0.023	2.74 (0.18)	2.12	1.66	0.064	0.054 - 0.075
MH-AG	2019	0.018	0.017 - 0.019	2.53 (0.14)	1.72	1.34	0.057	0.049 - 0.065
MH-AH	2019	0.019	0.018 - 0.020	2.55 (0.14)	1.87	1.46	0.061	0.053 - 0.071
MH-AK	2019	0.014	0.013 - 0.014	2.35 (0.12)	1.33	1.04	0.048	0.041 - 0.055
AP-KG	2019	0.015	0.014 - 0.016	2.48 (0.14)	1.43	1.12	0.048	0.041 - 0.056
AP-PB	2019	0.013	0.012 - 0.013	2.28 (0.12)	1.22	0.95	0.046	0.039 - 0.053
AP-WV	2019	0.011	0.011 - 0.012	2.77 (0.13)	1.10	0.86	0.033	0.029 - 0.037
KA-BA	2019	0.011	0.010 - 0.012	2.47 (0.24)	1.10	0.86	0.037	0.029 - 0.048
KA-BS	2019	0.009	0.009 - 0.010	2.22 (0.14)	0.89	0.70	0.035	0.029 - 0.041
KA-BI	2019	0.013	0.013 - 0.014	2.89 (0.16)	1.31	1.03	0.037	0.033 - 0.042
KA-BN	2019	0.021	0.020 - 0.022	2.71 (0.14)	2.06	1.61	0.063	0.055 - 0.071
KA-HA	2019	0.021	0.020 - 0.022	2.98 (0.19)	2.04	1.60	0.056	0.049 - 0.065
KA-RH	2019	0.013	0.012 - 0.014	2.17 (0.21)	1.24	0.97	0.050	0.037 - 0.066
TN-AR	2019	0.020	0.019 - 0.023	2.47 (0.26)	1.99	1.55	0.067	0.051 - 0.090
TN-AS1	2019	0.020	0.019 - 0.021	2.92 (0.14)	1.92	1.50	0.054	0.049 - 0.060
TN-AS2	2019	0.013	0.012 - 0.014	2.77 (0.15)	1.27	0.99	0.038	0.033 - 0.043
TN-CD	2019	0.025	0.023 - 0.027	2.57 (0.23)	2.45	1.92	0.079	0.063 - 0.100
TN-TA	2019	0.013	0.012 - 0.015	2.22 (0.23)	1.30	1.02	0.050	0.037 - 0.069
TN-TP	2019	0.009	0.008 - 0.010	1.61 (0.12)	0.91	0.71	0.058	0.043 - 0.079
PB-HS	2020	0.017	0.016 - 0.018	2.48 (0.13)	1.66	1.30	0.056	0.049 - 0.064
HR-KS	2020	0.020	0.019 - 0.022	2.02 (0.12)	1.99	1.56	0.088	0.073 - 0.107
MP-CC	2020	0.017	0.016 - 0.018	2.34 (0.15)	1.66	1.30	0.060	0.050 - 0.072
MP-CJ	2020	0.015	0.014 - 0.016	2.40 (0.14)	1.48	1.15	0.052	0.044 - 0.061
MP-KB	2020	0.016	0.016 - 0.017	2.22 (0.10)	1.60	1.25	0.062	0.054 - 0.071
MP-SK	2020	0.019	0.018 - 0.020	2.47 (0.12)	1.86	1.45	0.063	0.055 - 0.072
MP-ST	2020	0.017	0.017 - 0.018	2.46 (0.15)	1.70	1.33	0.058	0.049 - 0.067
MH-AK	2020	0.020	0.019 - 0.021	2.57 (0.13)	1.97	1.54	0.064	0.056 - 0.072
MH-AP	2020	0.019	0.018 - 0.020	2.37 (0.12)	1.82	1.43	0.065	0.056 - 0.075
AP-AD	2020	0.019	0.018 - 0.020	2.47 (0.11)	1.83	1.43	0.062	0.055 - 0.070
AP-ET	2020	0.013	0.012 - 0.014	2.23 (0.16)	1.29	1.01	0.050	0.040 - 0.061
AP-WV	2020	0.014	0.013 - 0.014	2.67 (0.18)	1.33	1.04	0.041	0.035 - 0.049
AP-PB	2020	0.013	0.013 - 0.014	2.82 (0.18)	1.28	1.00	0.037	0.032 - 0.043
KA-BI	2020	0.015	0.014 - 0.017	2.03 (0.13)	1.50	1.17	0.066	0.053 - 0.081
KA-DG	2020	0.021	0.020 - 0.022	2.50 (0.13)	2.06	1.61	0.069	0.060 - 0.079
KA-KV	2020	0.016	0.015 - 0.017	2.33 (0.14)	1.52	1.19	0.055	0.046 - 0.066
KA-RH	2020	0.020	0.019 - 0.022	2.03 (0.12)	1.99	1.56	0.087	0.072 - 0.106
TG-NV	2020	0.017	0.016 - 0.018	2.00 (0.14)	1.63	1.27	0.073	0.058 - 0.092
TN-CA	2020	0.027	0.025 - 0.028	2.57 (0.16)	2.61	2.04	0.085	0.072 - 0.099
TN-CM	2020	0.021	0.020 - 0.021	2.68 (0.10)	2.02	1.58	0.062	0.057 - 0.068
TN-ES	2020	0.019	0.018 - 0.020	2.58 (0.16)	1.85	1.45	0.060	0.051 - 0.070
TN-TT	2020	0.025	0.023 - 0.027	2.36 (0.18)	2.42	1.90	0.087	0.070 - 0.107
TN-TV	2020	0.027	0.026 - 0.028	2.61 (0.11)	2.61	2.04	0.083	0.074 - 0.092

[1]CI: confidence interval; [2]SE: standard error; [3]RR: resistance ratio, EC_{50} values of respective population divided by EC_{50} of population SUS-2005 or I-2018

Table S7. Log-dose mortality data for emamectin benzoate against third instar larvae of Indian populations of *Spodoptera frugiperda* in diet incorporation assays based on IRAC method no. 20. Mortality was assessed five days after treatment.

Sample ID	Year	EC_{50} [µg/mL]	95% CI[1]	Slope (±SE[2])	RR[3] to SUS-2005	RR[3] to I-2018	EC_{95} [µg/mL]	95% CI[1]
SUS-2005	2005	0.011	0.010 - 0.011	7.63 (1.21)	1.00	1.09	0.016	0.013 - 0.018
I-2018	2018	0.010	0.009 - 0.010	2.66 (0.14)	0.91	1.00	0.029	0.026 - 0.033
MP-BB	2019	0.012	0.012 - 0.013	2.37 (0.12)	1.15	1.25	0.042	0.037 - 0.048
MP-CC	2019	0.012	0.012 - 0.013	2.82 (0.17)	1.16	1.27	0.035	0.031 - 0.040
MP-CCD	2019	0.011	0.011 - 0.012	2.43 (0.15)	1.06	1.15	0.038	0.032 - 0.045
MP-IA	2019	0.012	0.012 - 0.013	2.27 (0.14)	1.16	1.26	0.045	0.038 - 0.053
MP-ID	2019	0.011	0.011 - 0.012	2.30 (0.13)	1.06	1.16	0.040	0.034 - 0.048
MP-SM	2019	0.011	0.010 - 0.011	2.44 (0.11)	1.00	1.09	0.036	0.031 - 0.040
MH-AG	2019	0.012	0.011 - 0.012	2.50 (0.15)	1.09	1.19	0.038	0.032 - 0.044
MH-AH	2019	0.013	0.012 - 0.014	2.33 (0.19)	1.20	1.32	0.045	0.036 - 0.057
MH-AK	2019	0.011	0.010 - 0.012	2.51 (0.14)	1.03	1.13	0.035	0.031 - 0.041
AP-KG	2019	0.010	0.009 - 0.010	2.61 (0.14)	0.91	0.99	0.030	0.026 - 0.034
AP-PB	2019	0.011	0.011 - 0.011	2.38 (0.10)	1.04	1.13	0.038	0.034 - 0.043
AP-WV	2019	0.012	0.012 - 0.013	2.64 (0.13)	1.15	1.26	0.037	0.033 - 0.042
KA-BA	2019	0.010	0.010 - 0.011	2.71 (0.13)	0.95	1.04	0.030	0.027 - 0.034
KA-BS	2019	0.012	0.011 - 0.013	2.62 (0.16)	1.13	1.24	0.037	0.032 - 0.043
KA-BI	2019	0.011	0.010 - 0.011	2.71 (0.15)	0.99	1.09	0.031	0.027 - 0.036
KA-BN	2019	0.008	0.008 - 0.008	2.30 (0.10)	0.76	0.83	0.029	0.026 - 0.033
KA-HA	2019	0.014	0.013 - 0.015	2.13 (0.13)	1.34	1.46	0.056	0.047 - 0.068
KA-RH	2019	0.011	0.011 - 0.012	2.40 (0.13)	1.07	1.17	0.039	0.033 - 0.044
TN-AR	2019	0.010	0.010 - 0.011	2.20 (0.12)	0.97	1.06	0.039	0.034 - 0.046
TN-AS1	2019	0.011	0.010 - 0.011	2.46 (0.09)	1.02	1.11	0.036	0.032 - 0.039
TN-AS2	2019	0.013	0.012 - 0.014	2.42 (0.15)	1.22	1.33	0.044	0.037 - 0.052
TN-CD	2019	0.013	0.013 - 0.014	2.18 (0.12)	1.26	1.37	0.051	0.044 - 0.060
TN-TA	2019	0.058	0.053 - 0.063	2.17 (0.18)	5.46	5.97	0.225	0.174 - 0.290
TN-TP	2019	0.011	0.011 - 0.012	1.92 (0.14)	1.08	1.18	0.053	0.042 - 0.068
PB-HS	2020	0.003	0.003 - 0.003	2.12 (0.11)	0.27	0.30	0.012	0.010 - 0.014
HR-KS	2020	0.013	0.013 - 0.014	2.59 (0.11)	1.23	1.35	0.041	0.037 - 0.045
MP-CC	2020	0.004	0.004 - 0.005	1.75 (0.10)	0.42	0.46	0.024	0.019 - 0.029
MP-CJ	2020	0.006	0.006 - 0.006	1.59 (0.08)	0.56	0.61	0.038	0.031 - 0.046
MP-KB	2020	0.004	0.004 - 0.004	2.35 (0.11)	0.38	0.41	0.014	0.012 - 0.016
MP-SK	2020	0.005	0.005 - 0.005	2.03 (0.10)	0.46	0.51	0.021	0.018 - 0.025
MP-ST	2020	0.004	0.004 - 0.004	2.35 (0.13)	0.39	0.43	0.014	0.012 - 0.017
MH-AK	2020	0.017	0.016 - 0.017	1.92 (0.09)	1.56	1.71	0.077	0.066 - 0.090
MH-AP	2020	0.022	0.021 - 0.023	1.97 (0.10)	2.09	2.29	0.099	0.083 - 0.117
AP-AD	2020	0.006	0.006 - 0.007	1.99 (0.14)	0.61	0.67	0.028	0.023 - 0.036
AP-ET	2020	0.005	0.005 - 0.006	2.55 (0.23)	0.51	0.56	0.017	0.014 - 0.022
AP-WV	2020	0.004	0.004 - 0.004	1.88 (0.12)	0.39	0.43	0.020	0.016 - 0.025
AP-PB	2020	0.006	0.006 - 0.007	2.18 (0.15)	0.58	0.63	0.024	0.019 - 0.029
KA-BI	2020	0.009	0.008 - 0.009	2.29 (0.12)	0.82	0.89	0.031	0.027 - 0.037
KA-DG	2020	0.006	0.005 - 0.006	2.40 (0.13)	0.52	0.57	0.019	0.016 - 0.022
KA-KV	2020	0.006	0.006 - 0.007	2.29 (0.13)	0.60	0.66	0.023	0.020 - 0.027
KA-RH	2020	0.007	0.006 - 0.007	1.86 (0.14)	0.62	0.67	0.032	0.024 - 0.041
TG-NV	2020	0.012	0.011 - 0.012	1.89 (0.11)	1.09	1.20	0.055	0.045 - 0.068
TN-CA	2020	0.006	0.006 - 0.006	1.97 (0.13)	0.56	0.62	0.027	0.021 - 0.033
TN-CM	2020	0.014	0.013 - 0.015	2.00 (0.11)	1.32	1.44	0.061	0.051 - 0.073
TN-ES	2020	0.005	0.005 - 0.005	2.37 (0.11)	0.48	0.52	0.018	0.015 - 0.020
TN-TT	2020	0.008	0.008 - 0.009	2.18 (0.12)	0.80	0.88	0.033	0.028 - 0.039

TN-TV	2020	0.012	0.011 - 0.013	2.58 (0.17)	1.11	1.22	0.037	0.031 - 0.044

[1]CI: confidence interval; [2]SE: standard error; [3]RR: resistance ratio, EC_{50} values of respective population divided by EC_{50} of population SUS-2005 or I-2018

Table S8. Log-dose mortality data for flubendiamide against third instar larvae of Indian populations of *Spodoptera frugiperda* in diet incorporation assays based on IRAC method no. 20. Mortality was assessed five days after treatment.

Sample ID	Year	EC_{50} [µg/mL]	95% CI[1]	Slope (±SE[2])	RR[3] to SUS-2005	RR[3] to I-2018	EC_{95} [µg/mL]	95% CI[1]
SUS-2005	2005	0.034	0.033 - 0.035	4.59 (0.26)	1.00	0.30	0.065	0.060 - 0.071
I-2018	2018	0.114	0.109 - 0.120	2.30 (0.12)	3.33	1.00	0.412	0.357 - 0.476
MP-BB	2019	0.224	0.214 - 0.234	2.29 (0.10)	6.51	1.96	0.811	0.716 - 0.919
MP-CC	2019	0.212	0.205 - 0.220	2.59 (0.10)	6.17	1.85	0.661	0.597 - 0.731
MP-CCD	2019	0.238	0.224 - 0.253	2.28 (0.14)	6.92	2.08	0.865	0.727 - 1.029
MP-IA	2019	0.748	0.702 - 0.797	2.14 (0.13)	21.79	6.55	2.956	2.458 - 3.555
MP-ID	2019	0.520	0.503 - 0.539	2.54 (0.10)	15.15	4.55	1.659	1.502 - 1.831
MP-SM	2019	0.249	0.240 - 0.258	2.62 (0.11)	7.25	2.18	0.767	0.693 - 0.849
MH-AG	2019	0.146	0.137 - 0.155	2.31 (0.15)	4.24	1.27	0.521	0.436 - 0.622
MH-AH	2019	0.102	0.099 - 0.105	3.01 (0.13)	2.98	0.89	0.272	0.248 - 0.298
MH-AK	2019	0.113	0.108 - 0.118	2.40 (0.11)	3.30	0.99	0.387	0.341 - 0.439
AP-KG	2019	0.223	0.216 - 0.231	2.54 (0.10)	6.50	1.95	0.711	0.643 - 0.785
AP-PB	2019	0.264	0.252 - 0.278	2.34 (0.12)	7.70	2.31	0.930	0.807 - 1.071
AP-WV	2019	0.241	0.230 - 0.254	2.48 (0.13)	7.03	2.11	0.792	0.690 - 0.909
KA-BA	2019	0.294	0.279 - 0.310	3.04 (0.21)	8.56	2.57	0.775	0.667 - 0.901
KA-BS	2019	0.298	0.286 - 0.311	2.94 (0.16)	8.68	2.61	0.812	0.722 - 0.914
KA-BI	2019	0.568	0.533 - 0.606	2.68 (0.20)	16.55	4.97	1.706	1.423 - 2.047
KA-BN	2019	0.511	0.482 - 0.542	2.06 (0.11)	14.87	4.47	2.127	1.791 - 2.525
KA-HA	2019	0.841	0.792 - 0.894	2.31 (0.14)	24.50	7.36	3.007	2.525 - 3.581
KA-RH	2019	0.276	0.252 - 0.301	1.82 (0.14)	8.03	2.41	1.391	1.062 - 1.823
TN-AR	2019	0.101	0.093 - 0.110	1.89 (0.15)	2.95	0.89	0.480	0.370 - 0.622
TN-AS1	2019	0.451	0.428 - 0.475	2.32 (0.12)	13.13	3.95	1.602	1.379 - 1.860
TN-AS2	2019	0.436	0.407 - 0.467	2.20 (0.15)	12.69	3.81	1.658	1.359 - 2.023
TN-CD	2019	0.310	0.290 - 0.332	2.22 (0.15)	9.03	2.71	1.169	0.959 - 1.424
TN-TA	2019	0.273	0.252 - 0.296	1.95 (0.14)	7.95	2.39	1.239	0.974 - 1.577
TN-TP	2019	0.375	0.359 - 0.392	2.44 (0.12)	10.91	3.28	1.252	1.102 - 1.423
PB-HS	2020	0.633	0.590 - 0.678	2.07 (0.15)	18.42	5.53	2.616	2.005 - 3.412
HR-KS	2020	0.442	0.409 - 0.478	2.34 (0.19)	12.87	3.87	1.556	1.235 - 1.961
MP-CC	2020	0.308	0.287 - 0.330	1.94 (0.12)	8.96	2.69	1.401	1.137 - 1.726
MP-CJ	2020	0.308	0.287 - 0.330	1.94 (0.12)	8.96	2.69	1.401	1.137 - 1.726
MP-KB	2020	0.241	0.229 - 0.254	2.33 (0.12)	7.02	2.11	0.856	0.738 - 0.992
MP-SK	2020	0.354	0.336 - 0.372	2.57 (0.15)	10.29	3.09	1.110	0.958 - 1.286
MP-ST	2020	0.390	0.369 - 0.414	2.03 (0.11)	11.37	3.42	1.664	1.395 - 1.985
MH-AK	2020	0.122	0.116 - 0.129	1.95 (0.10)	3.56	1.07	0.552	0.467 - 0.654
MH-AP	2020	0.076	0.070 - 0.082	1.60 (0.10)	2.20	0.66	0.477	0.367 - 0.620
AP-AD	2020	0.275	0.254 - 0.298	2.03 (0.15)	8.02	2.41	1.171	0.929 - 1.478
AP-ET	2020	0.056	0.052 - 0.061	2.22 (0.17)	1.63	0.49	0.212	0.168 - 0.266
AP-WV	2020	0.040	0.037 - 0.043	2.24 (0.18)	1.17	0.35	0.150	0.118 - 0.189
AP-PB	2020	0.108	0.101 - 0.117	2.21 (0.16)	3.15	0.95	0.410	0.329 - 0.512
KA-BI	2020	0.623	0.585 - 0.663	2.03 (0.12)	18.14	5.45	2.651	2.204 - 3.188
KA-DG	2020	0.287	0.270 - 0.305	2.17 (0.13)	8.35	2.51	1.112	0.929 - 1.330
KA-KV	2020	0.373	0.354 - 0.393	2.52 (0.15)	10.86	3.26	1.201	1.032 - 1.398
KA-RH	2020	0.222	0.211 - 0.234	2.12 (0.11)	6.47	1.94	0.892	0.763 - 1.041
TG-NV	2020	0.776	0.715 - 0.843	1.85 (0.13)	22.61	6.79	3.799	2.959 - 4.878
TN-CA	2020	0.195	0.183 - 0.208	2.35 (0.16)	5.68	1.71	0.683	0.566 - 0.825
TN-CM	2020	0.151	0.140 - 0.163	1.84 (0.12)	4.39	1.32	0.746	0.593 - 0.939
TN-ES	2020	0.134	0.128 - 0.140	2.56 (0.13)	3.90	1.17	0.423	0.372 - 0.481

TN-TT	2020	0.561	0.525 - 0.598	1.93 (0.11)	16.32	4.90	2.578	2.124 - 3.129
TN-TV	2020	0.723	0.665 - 0.787	2.12 (0.17)	21.06	6.33	2.899	2.257 - 3.724

[1]CI: confidence interval; [2]SE: standard error; [3]RR: resistance ratio, EC_{50} values of respective population divided by EC_{50} of population SUS-2005 or I-2018

Table S9. Log-dose mortality data for chlorantraniliprole against third instar larvae of Indian populations of *Spodoptera frugiperda* in diet incorporation assays based on IRAC method no. 20. Mortality was assessed five days after treatment.

Sample ID	Year	EC_{50} [μg/mL]	95% CI[1]	Slope (±SE[2])	RR[3] to SUS-2005	RR[3] to I-2018	EC_{95} [μg/mL]	95% CI[1]
SUS-2005	2005	0.005	0.005 – 0.006	4.54 (0.76)	1.00	0.60	0.010	0.008 - 0.012
I-2018	2018	0.009	0.008 – 0.009	2.39 (0.11)	1.68	1.00	0.029	0.026 - 0.033
PB-HS	2020	0.016	0.015 - 0.017	1.63 (0.08)	3.18	1.90	0.098	0.080 - 0.120
HR-KS	2020	0.035	0.033 - 0.037	2.12 (0.11)	6.84	4.08	0.139	0.118 - 0.164
MP-CC	2020	0.012	0.011 - 0.013	2.04 (0.11)	2.40	1.43	0.052	0.043 - 0.062
MP-CJ	2020	0.028	0.026 - 0.031	1.38 (0.07)	5.53	3.30	0.236	0.184 - 0.304
MP-KB	2020	0.026	0.025 - 0.028	2.12 (0.12)	5.18	3.09	0.106	0.089 - 0.125
MP-SK	2020	0.012	0.012 - 0.013	2.36 (0.14)	2.44	1.46	0.043	0.036 - 0.051
MP-ST	2020	0.010	0.009 - 0.011	1.31 (0.08)	2.01	1.20	0.096	0.071 - 0.132
MH-AK	2020	0.018	0.017 - 0.018	2.29 (0.10)	3.47	2.07	0.064	0.057 - 0.072
MH-AP	2020	0.017	0.016 - 0.018	2.37 (0.10)	3.34	2.00	0.059	0.052 - 0.066
AP-AD	2020	0.007	0.007 - 0.007	1.82 (0.08)	1.37	0.82	0.035	0.030 - 0.042
AP-ET	2020	0.020	0.020 - 0.021	2.46 (0.12)	4.03	2.40	0.068	0.059 - 0.077
AP-WV	2020	0.005	0.005 - 0.005	1.69 (0.11)	0.96	0.57	0.028	0.022 - 0.036
AP-PB	2020	0.020	0.019 - 0.021	2.14 (0.12)	3.93	2.35	0.079	0.066 - 0.094
KA-BI	2020	0.024	0.022 - 0.025	2.14 (0.14)	4.66	2.78	0.094	0.076 - 0.115
KA-DG	2020	0.024	0.022 - 0.026	2.09 (0.14)	4.70	2.81	0.097	0.079 - 0.121
KA-KV	2020	0.013	0.012 - 0.013	2.55 (0.14)	2.50	1.49	0.040	0.035 - 0.046
KA-RH	2020	0.010	0.009 - 0.011	1.74 (0.12)	1.95	1.16	0.054	0.042 - 0.070
TG-NV	2020	0.023	0.021 - 0.025	1.98 (0.14)	4.49	2.68	0.101	0.080 - 0.127
TN-CA	2020	0.017	0.017 - 0.018	2.14 (0.09)	3.43	2.05	0.069	0.060 - 0.079
TN-CM	2020	0.020	0.019 - 0.022	1.90 (0.12)	3.95	2.36	0.095	0.076 - 0.118
TN-ES	2020	0.018	0.017 - 0.019	2.23 (0.11)	3.48	2.08	0.066	0.057 - 0.077
TN-TT	2020	0.005	0.005 - 0.006	2.30 (0.14)	1.03	0.61	0.019	0.016 - 0.022
TN-TV	2020	0.005	0.004 - 0.005	2.08 (0.12)	0.91	0.54	0.019	0.016 - 0.023

[1]CI: confidence interval; [2]SE: standard error; [3]RR: resistance ratio, EC_{50} values of respective population divided by EC_{50} of population SUS-2005 or I-2018

Table S10. Log-dose mortality data for tetraniliprole against third instar larvae of Indian populations of *Spodoptera frugiperda* in diet incorporation assays based on IRAC method no. 20. Mortality was assessed five days after treatment.

Sample ID	Year	EC_{50} [µg/mL]	95% CI[1]	Slope (±SE[2])	RR[3] to SUS-2005	RR[3] to I-2018	EC_{95} [µg/mL]	95% CI[1]
SUS-2005	2005	0.016	0.015 - 0.016	4.42 (0.25)	1.00	0.79	0.030	0.028 - 0.033
I-2018	2018	0.020	0.019 - 0.021	2.54 (0.15)	1.27	1.00	0.063	0.054 - 0.073
MP-BB	2019	0.018	0.017 - 0.018	2.61 (0.12)	1.14	0.90	0.055	0.049 - 0.061
MP-CC	2019	0.016	0.015 - 0.017	2.42 (0.16)	1.03	0.81	0.054	0.045 - 0.065
MP-CCD	2019	0.013	0.012 - 0.014	2.63 (0.19)	0.83	0.65	0.039	0.033 - 0.047
MP-IA	2019	0.013	0.012 - 0.014	2.14 (0.11)	0.84	0.66	0.052	0.044 - 0.060
MP-ID	2019	0.013	0.012 - 0.014	2.10 (0.13)	0.82	0.64	0.052	0.043 - 0.063
MP-SM	2019	0.011	0.011 - 0.012	2.34 (0.11)	0.73	0.58	0.040	0.035 - 0.046
MH-AG	2019	0.016	0.015 - 0.017	2.27 (0.14)	1.02	0.80	0.058	0.049 - 0.069
MH-AH	2019	0.015	0.014 - 0.016	2.51 (0.15)	0.96	0.76	0.048	0.041 - 0.056
MH-AK	2019	0.012	0.012 - 0.013	2.29 (0.10)	0.78	0.61	0.044	0.038 - 0.050
AP-KG	2019	0.013	0.013 - 0.014	2.60 (0.13)	0.86	0.68	0.042	0.037 - 0.047
AP-PB	2019	0.017	0.016 - 0.018	2.16 (0.14)	1.07	0.84	0.065	0.054 - 0.079
AP-WV	2019	0.021	0.020 - 0.022	2.30 (0.12)	1.36	1.07	0.076	0.065 - 0.088
KA-BA	2019	0.015	0.014 - 0.015	2.27 (0.11)	0.93	0.73	0.053	0.046 - 0.061
KA-BS	2019	0.019	0.018 - 0.020	2.95 (0.18)	1.24	0.98	0.052	0.046 - 0.060
KA-BI	2019	0.018	0.017 - 0.019	2.59 (0.15)	1.14	0.90	0.055	0.047 - 0.064
KA-BN	2019	0.023	0.022 - 0.024	2.22 (0.13)	1.47	1.15	0.086	0.073 - 0.101
KA-HA	2019	0.014	0.013 - 0.014	2.26 (0.09)	0.89	0.70	0.051	0.045 - 0.058
KA-RH	2019	0.022	0.021 - 0.024	2.42 (0.16)	1.43	1.13	0.075	0.063 - 0.090
TN-AR	2019	0.020	0.019 - 0.022	2.37 (0.15)	1.31	1.03	0.071	0.060 - 0.084
TN-AS1	2019	0.017	0.015 - 0.018	2.18 (0.15)	1.06	0.84	0.064	0.052 - 0.078
TN-AS2	2019	0.020	0.019 - 0.022	2.20 (0.14)	1.30	1.03	0.077	0.064 - 0.093
TN-CD	2019	0.024	0.023 - 0.025	2.82 (0.16)	1.52	1.20	0.067	0.059 - 0.077
TN-TA	2019	0.021	0.020 - 0.022	2.39 (0.12)	1.37	1.08	0.073	0.064 - 0.084
TN-TP	2019	0.018	0.016 - 0.019	2.36 (0.22)	1.13	0.89	0.061	0.047 - 0.078
PB-HS	2020	0.008	0.007 - 8424	2.15 (0.13)	0.51	0.40	0.031	0.026 - 0.037
HR-KS	2020	0.034	0.033 - 0.036	2.52 (0.15)	2.21	1.74	0.110	0.095 - 0.129
MP-CC	2020	0.019	0.018 - 0.021	2.14 (0.14)	1.25	0.98	0.077	0.062 - 0.094
MP-CJ	2020	0.015	0.015 - 0.016	2.55 (0.10)	0.97	0.77	0.048	0.043 - 0.053
MP-KB	2020	0.022	0.021 - 0.023	1.95 (0.08)	1.41	1.11	0.099	0.086 - 0.115
MP-SK	2020	0.023	0.021 - 0.025	1.92 (0.12)	1.48	1.17	0.107	0.085 - 0.133
MP-ST	2020	0.021	0.020 - 0.021	2.35 (0.09)	1.32	1.04	0.072	0.064 - 0.081
MH-AK	2020	0.015	0.015 - 0.016	1.96 (0.09)	0.99	0.78	0.069	0.060 - 0.080
MH-AP	2020	0.013	0.012 - 0.014	1.35 (0.08)	0.84	0.66	0.114	0.088 - 0.149
AP-AD	2020	0.028	0.027 - 0.030	2.69 (0.14)	1.83	1.44	0.085	0.075 - 0.096
AP-ET	2020	0.017	0.015 - 0.018	2.52 (0.22)	1.08	0.85	0.054	0.043 - 0.067
AP-WV	2020	0.013	0.012 - 0.013	2.23 (0.13)	0.80	0.63	0.047	0.040 - 0.055
AP-PB	2020	0.019	0.019 - 0.020	2.76 (0.14)	1.25	0.99	0.057	0.050 - 0.064
KA-BI	2020	0.025	0.024 - 0.027	2.24 (0.12)	1.64	1.29	0.095	0.081 - 0.110
KA-DG	2020	0.018	0.017 - 0.019	2.39 (0.16)	1.14	0.90	0.061	0.050 - 0.074
KA-KV	2020	0.019	0.019 - 0.020	2.56 (0.14)	1.25	0.99	0.062	0.054 - 0.071
KA-RH	2020	0.023	0.021 - 0.025	2.11 (0.17)	1.49	1.17	0.093	0.073 - 0.120
TG-NV	2020	0.029	0.027 - 0.032	1.84 (0.13)	1.87	1.47	0.144	0.112 - 0.186
TN-CA	2020	0.034	0.031 - 0.036	2.27 (0.18)	2.17	1.71	0.123	0.098 - 0.156

TN-CM	2020	0.018	0.018 - 0.019	2.60 (0.13)	1.19	0.93	0.057	0.050 - 0.065
TN-ES	2020	0.036	0.034 - 0.038	1.88 (0.09)	2.30	1.81	0.171	0.144 - 0.203
TN-TT	2020	0.022	0.021 - 0.023	2.13 (0.12)	1.41	1.11	0.087	0.074 - 0.103
TN-TV	2020	0.022	0.020 - 0.023	1.95 (0.13)	1.40	1.10	0.098	0.079 - 0.123

[1]CI: confidence interval; [2]SE: standard error; [3]RR: resistance ratio, EC_{50} values of respective population divided by EC_{50} of population SUS-2005 or I-2018

Table S11. Composite of EC_{50} / EC_{95} values of *Spodoptera frugiperda* populations treated with chlorpyrifos, deltamethrin and flubendiamide insecticides comparing collection years.

Compound	Year	No. Populations	EC_{50} [μg/mL]	95% CI	Slope (± SE)	EC_{95} [μg/mL]	95% CI
Chlorpyrifos	SUS-2005	1	1.783	1.693 - 1.879	6.72 (0.95)	2.763	2.419 - 3.157
	I-2018	1	7.509	7.119 – 7.921	2.31 (0.12)	26.870	23.040 – 31.330
	2019	7	5.562	5.297 – 5.841	2.09 (0.10)	22.780	19.710 - 26.340
	2020	23	6.960	6.575 - 7.358	1.50 (0.07)	49.590	40.980 - 60.010
Deltamethrin	SUS-2005	1	0.198	0.190 - 0.206	2.94 (0.16)	0.538	0.481 - 0.602
	I-2018	1	4.271	3.946 - 4.623	2.12 (0.16)	17.080	13.420 - 21.750
	2019	7	3.347	3.101 - 3.612	1.74 (0.12)	18.160	14.210 - 23.210
	2020	23	4.931	4.734 - 5.136	1.84 (0.07)	24.500	21.580 - 27.830
Flubendiamide	SUS-2005	1	0.034	0.033 - 0.035	4.59 (0.26)	0.065	0.060 - 0.071
	I-2018	1	0.114	0.109 - 0.120	2.30 (0.12)	0.412	0.357 – 0.476
	2019	24	0.277	0.261 - 0.294	1.59 (0.08)	1.772	1.467 - 2.142
	2020	23	0.272	0.247 - 0.299	1.06 (0.07)	4.395	3.064 - 6.305

[1]CI: confidence interval; [2]SE: standard error
